# Mathematical modeling of mechanosensitive reversal control in *Myxococcus xanthus*

**DOI:** 10.3389/fmicb.2023.1294631

**Published:** 2024-01-08

**Authors:** Yirui Chen, Elias J. Topo, Beiyan Nan, Jing Chen

**Affiliations:** ^1^Department of Biological Sciences, Virginia Tech, Blacksburg, VA, United States; ^2^Genetics, Bioinformatics and Computational Biology Graduate Program, Virginia Tech, Blacksburg, VA, United States; ^3^Department of Biology, Texas A&M University, College Station, TX, United States; ^4^Fralin Life Sciences Institute, Virginia Tech, Blacksburg, VA, United States; ^5^Center for Soft Matter and Biological Physics, Virginia Tech, Blacksburg, VA, United States

**Keywords:** mechanosensing, bacterial motility, gliding motility, polarity regulation, myxobacteria, mathematical modeling

## Abstract

Adjusting motility patterns according to environmental cues is important for bacterial survival. *Myxococcus xanthus*, a bacterium moving on surfaces by gliding and twitching mechanisms, modulates the reversal frequency of its front-back polarity in response to mechanical cues like substrate stiffness and cell-cell contact. In this study, we propose that *M. xanthus*’s gliding machinery senses environmental mechanical cues during force generation and modulates cell reversal accordingly. To examine our hypothesis, we expand an existing mathematical model for periodic polarity reversal in *M. xanthus*, incorporating the experimental data on the intracellular dynamics of the gliding machinery and the interaction between the gliding machinery and a key polarity regulator. The model successfully reproduces the dependence of cell reversal frequency on substrate stiffness observed in *M. xanthus* gliding. We further propose reversal control networks between the gliding and twitching motility machineries to explain the opposite reversal responses observed in wild type *M. xanthus* cells that possess both motility mechanisms. These results provide testable predictions for future experimental investigations. In conclusion, our model suggests that the gliding machinery in *M. xanthus* can function as a mechanosensor, which transduces mechanical cues into a cell reversal signal.

## Introduction

The ability to sense the environment and adapt their behaviors accordingly is crucial for survival and propagation of bacteria. For motile bacteria, their motility is often highly adaptable to environmental cues. A well-studied example is chemotaxis in *Escherichia coli* swimming: the bacterium decreases its tumbling frequency when it senses an increase of nutrient concentration, and vice versa (Berg and Brown, 1972). Over a long time scale, this response amounts to biased cell movements toward nutrient-dense areas (Wadhams and Armitage, 2004; Sourjik and Wingreen, 2012). Besides diffusive chemical signals, bacteria can also adapt their motility (and other behaviors) to mechanical cues (Persat et al., 2015; Colin et al., 2019; Gordon and Wang, 2019; Dufrene and Persat, 2020). For instance, increasing the load on an *E. coli* flagellum stabilizes the stator units in the flagellar motor, leading to an increased power output and recovery of the rotation frequency of the flagellum (Lele et al., 2013; Tipping et al., 2013; Wadhwa et al., 2019). Moreover, Type IV pilus, an appendage driving twitching motility in a broad range of bacteria, switches from extension to retraction rapidly and nearly exclusively upon touching a surface with its tip, suggesting that it can sense physical contacts (Tala et al., 2019; Dufrene and Persat, 2020). Because bacteria experience constantly fluctuating forces, especially when they live on inhomogeneous surfaces or in complex biofilms, their ability to respond to mechanical cues plays a critical role in their survival.

In this work, we focus on the cellular mechanism for mechanosensing in the motility of a soil-dwelling bacterium, *Myxococcus xanthus*. *M. xanthus* is a model organism for studying bacterial social behaviors, as it features complex spatial patterns and structures at the population level, such as streams, rippling waves, aggregation and fruiting bodies (Welch and Kaiser, 2001; Zhang et al., 2012a; Keane and Berleman, 2016). These spatial patterns and structures play important roles in “social” collaboration during predation and sporulation (Berleman and Kirby, 2009; Velicer and Vos, 2009; Zhang et al., 2012a; Munoz-Dorado et al., 2016). Formation of the spatial patterns and structures hinges on the *M. xanthus* cells’ ability to modulate their motility in response to external stimuli (Berleman and Kirby, 2007). Specifically, *M. xanthus* frequently reverses its direction of motion (Mauriello et al., 2010a; Schumacher and Sogaard-Andersen, 2017) and its reversal frequency is influenced by both chemical and mechanical cues (Igoshin et al., 2001, 2004; Borner et al., 2002; Kaimer et al., 2012). For the latter, the reversal frequency changes upon physical contact with other *M. xanthus* cells (Shi et al., 1996; Welch and Kaiser, 2001) or prey cells (Mcbride and Zusman, 1996; Zhang et al., 2020), and varies with substrates stiffness (Zhou and Nan, 2017). In this work, as a starting point to dissect the cellular mechanism of *M. xanthus* mechanosensing, we focused on investigating the dependence of the cell reversal frequency on substrate stiffness.

*M. xanthus* moves on surfaces through social (S)-motility favored by cells in large groups and adventurous (A)-motility favored by isolated cells (Shi and Zusman, 1993). S-motility, also known as twitching motility, is driven by Type IV pili (Sun et al., 2000; Mauriello et al., 2010a; Zhang et al., 2012a), the same cellular appendage that drives the twitching motility in *Pseudomonas aeruginosa* (Burrows, 2012). A-motility, also known as gliding motility, is powered by multi-subunit Agl-Glt complexes, which actively travel along helical intracellular trajectories and generate propulsion as they aggregate in the so-called focal adhesion sites that contact the underlying substrate (Nan et al., 2011, 2013, 2014; Islam and Mignot, 2015; Faure et al., 2016). Both the S- and A-motility machineries are activated at the leading pole of the cell (Schumacher and Sogaard-Andersen, 2017; Carreira et al., 2022). The polarity of the cell is defined by asymmetric concentration of polarity regulators. Particularly, the Ras-like GTPase MglA (Mauriello et al., 2010b) concentrates at the leading pole, while its cognate GTPase activating protein, the MglB/RomY complex (Miertzschke et al., 2011; Szadkowski et al., 2022), and cognate guanine nucleotide exchange factor, the RomR/RomX complex (Szadkowski et al., 2019), concentrate at the trailing pole (Leonardy et al., 2007, 2010; Patryn et al., 2010; Zhang et al., 2010; Bulyha et al., 2011). These polarity regulators form feedback loops that amount to a spatial oscillator where the regulators periodically switch between the two cell poles and reverse the cell’s polarity and direction of motion (Zhang et al., 2010, 2012b; Treuner-Lange et al., 2015; Schumacher and Sogaard-Andersen, 2017; Guzzo et al., 2018; Szadkowski et al., 2019; Carreira et al., 2022). Surprisingly, wild type *M. xanthus* cells reverse on hard, 1.5% agar almost twice as frequently as they do on soft, 0.5% agar (Zhou and Nan, 2017), while the opposite trend was observed in *M. xanthus* mutants without the S-motility (S^−^ cells) (this work).

Although the molecular mechanisms for force generation (Sun et al., 2000, 2011; Mignot et al., 2007; Nan et al., 2011, 2013; Faure et al., 2016) and polarity switching (Zhang et al., 2010, 2012b; Guzzo et al., 2018; Galicia et al., 2019; Szadkowski et al., 2019, 2022) have been intensively studied in *M. xanthus*, it remains unknown how mechanosensing regulates cell reversal. Mechanosensing relies on molecules capable of converting external mechanical cues into intracellular signals. Among the most promising candidates playing this role are motility machineries, which, by the nature of their function, form mechanical links between the cell and external substrates. In eukaryotes, for example, the focal adhesion mechanism (macromolecular structures that dynamically assemble and drive cell migration) indeed mediates mechanosensing through mechanical connection to the extracellular matrix (Cheng et al., 2017; Tao et al., 2017; Martino et al., 2018). In *M. xanthus*, the polarity regulator MglA is also an essential subunit of the active A-motility machinery (Patryn et al., 2010; Zhang et al., 2010; Treuner-Lange et al., 2015; Faure et al., 2016). We hence hypothesize that the A-motility machinery may influence the polarity pathway and modulate cell reversal in response to external mechanical cues.

To investigate our hypothesis, we expanded an existing mathematical model for periodic polarity switch in *M. xanthus* (Guzzo et al., 2018), and incorporated the experimental data on the subcellular dynamics of A-motility machineries and the interaction between MglA and the A-motility machinery. The model-predicted relationship between substrate stiffness and cell reversal frequency is consistent with the experimental observation in S^−^ mutant cells with only A-motility. To elucidate the opposite dependence of reversal frequency on substrate stiffness observed in wild type cells with both A-and S-motility, we further examined extended pathways in which the S-motility machinery influences cell reversal as well. Because direct link between the S-motility machinery and the polarity pathway is yet missing and the reversal frequency of cells that moves with S-motility alone is insensitive to substrate stiffness (Zhou and Nan, 2017), we assumed that the S-motility machinery affects cell reversal indirectly through the A-motility machinery, and only examined generic pathways within this category. For each candidate pathway, we combined its topology with our model results and qualitatively predicted the dependence of cell reversal frequency on substrate stiffness in both genetic backgrounds. The qualitative predictions suggest that promotion of the activation of A-motility machinery by S-motility is necessary to reconcile the opposite reversal responses in wild type cells vs. S^−^ mutants. Our model proposes a mechanosensing mechanism through A-motility in *M. xanthus* and provides testable predictions for future experimental study.

## Results

### Modeling the coupling between polarity control and A-motility

**Figure 1:**
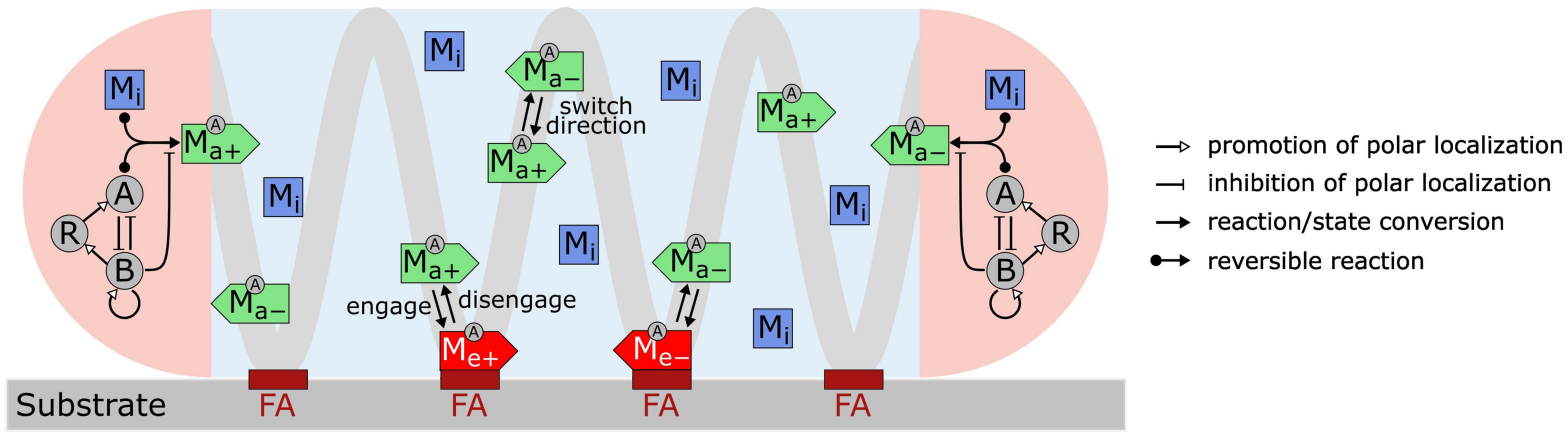
Model for *M. xanthus* reversal control with mechanosensing through A-motors. A-motors are present in three states in the cell: inactive (M_i_), active (M_a+_, M_a−_) and engaged (M_e+_, M_e−_). Inactive motors diffuse in the cell. Active motors move directionally along the helical trajectory (light gray line) and randomly switch directions (+: moving to the right; −: moving to the left). When active motors pass a focal adhesion site (maroon bars labeled as “FA”), they can become engaged in force generation. Activation and deactivation of motors only occur at the cell poles (pink areas). A motor is activated through binding with MglA (A) and is inactivated by MglB (B). Feedback loops among MglA (A), MglB (B) and RomR (R), as proposed by Guzzo et al. (2018), control periodic polarity switch.

We constructed a model (Figure 1) that incorporates the following key experimental observations. For short, we refer to the multi-subunit A-motility machinery as the “A-motor.”

1. An A-motor can assume three possible states: inactive, active, or engaged. An inactive motor, which likely represents disassembled parts of the motor (Nan et al., 2015; Faure et al., 2016; Nan, 2017), only diffuses in the cell.
2. An active motor travels along the helical track towards either the leading or trailing pole. The directionality of an active motor randomly switches (Nan et al., 2015), probably due to the lack of polarity in the short patches of MreB filaments (Kim et al., 2006; Van Den Ent et al., 2014; Errington, 2015; Billaudeau et al., 2017), on which the A-motor moves along (Mauriello et al., 2010b; Nan et al., 2011; Treuner-Lange et al., 2015; Fu et al., 2018).
3. As an active A-motor passes through a focal adhesion site, it can engage with the focal adhesion site and generate thrust. The engagement of a motor at the focal adhesion site likely represents coupling between the inner-membrane energy-harvesting subunits and the periplasmic and outer-membrane subunits (Islam et al., 2023).
4. Activation and deactivation of A-motors occur at the cell poles, where the polarity regulators are concentrated (Zhang et al., 2010, 2012b; Treuner-Lange et al., 2015; Guzzo et al., 2018; Galicia et al., 2019; Szadkowski et al., 2019, 2022). The motor is activated upon binding with MglA, which is reversed by MglB, the cognate GTPase activating protein for MglA (Miertzschke et al., 2011).
5. The polarity pathway follows a recent model (Guzzo et al., 2018). Specifically, MglA and MglB antagonize each other in polar localization; MglB promotes polar localization of RomR and its own polar localization; finally, RomR promotes polar localization of MglA. Strong mutual inhibition between MglA and MglB breaks the symmetry and makes them concentrate at opposite poles. The negative feedback loop, MglA ⊣ MglB → RomR → MglA, causes periodic switching in their polar localization.

**Figure 2:**
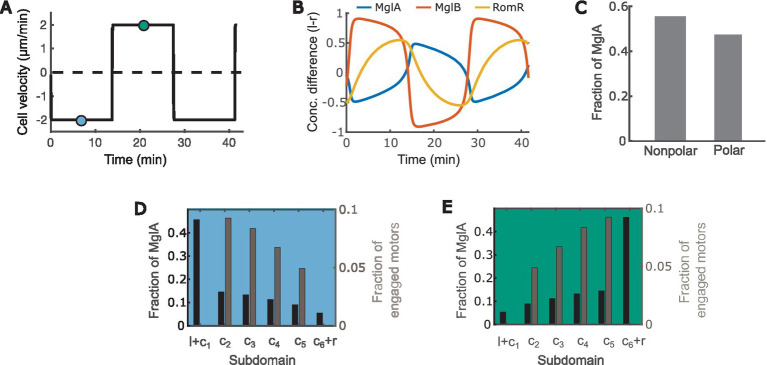
The model recapitulates key characteristics of *M. xanthus* motility. **(A)** Time course of the cell velocity. Positive and negative velocities correspond to motion towards the right and left, respectively. **(B)** Time courses of the concentration difference of MglA (A), MglB (B) and RomR (R) between the left (l) and right (r) poles. Positive and negative values indicate concentrations of the molecules at the left and right poles, respectively. **(C)** Distribution of MglA in the polar vs. nonpolar domains at the steady phase of the reversal cycle. Data sampled at the teal and/or blue points in **(A)**. **(D,E)** Spatial distribution of engaged motors and MglA along the cell when the cell is heading right **(D)** vs. left **(E)**. Subdomain labels: l: bound to the left pole. r: bound to the right pole. c_1_: unbound, in cytoplasmic domain at the left pole. c_6_: unbound, in cytoplasmic domain at the right pole. c_2 ~ 5_: in cytoplasmic subdomains between poles. **(D,E)** present data sampled at the teal and blue time points, respectively, in **(A)**.

With the equations and parameters given in Supplementary Methods (Supplementary Equations S1–S32; Supplementary Tables S1–S3), our model recapitulates key motility characteristics, including:

1. The cell reverses periodically every ~12 min (Figure 2A) (Kaimer and Zusman, 2016; Guzzo et al., 2018; Szadkowski et al., 2019).
2. MglA concentrates at the leading pole, and MglB and RomR concentrate at the trailing pole (Figure 2B) (Zhang et al., 2010, 2012b; Treuner-Lange et al., 2015; Guzzo et al., 2018; Szadkowski et al., 2019).
3. MglA forms a concentration gradient between the leading and trailing cell poles (Figures 2D,E), and about half of the MglA molecules are localized outside the polar regions (Figure 2C) (Nan et al., 2015).
4. Active motors form a concentration gradient that decreases from the leading pole to the trailing pole (Figures 2D,E) (Mignot et al., 2007).

### Interaction between MglA and A-motors predicts dependence of cell reversal frequency on substrate stiffness in A^+^S^−^ cells

**Figure 3:**
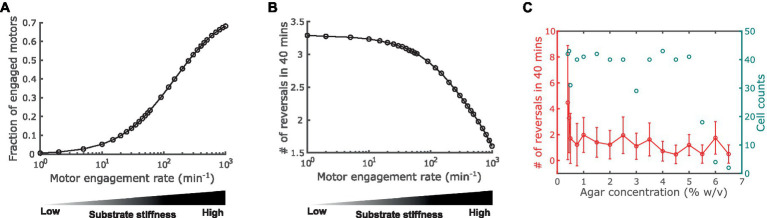
Impact of substrate stiffness on cell reversal frequency. **(A)** Using A-motor engagement rate as a proxy for substrate stiffness in the model. Harder substrates are represented by higher engagement rates, which result in higher fractions of engaged A-motors. The model parameters are tuned such that the range of the fraction of engaged A-motors is consistent with the experimental observation in Nan et al. (2013). **(B)** Model predicted dependence of the cell reversal frequency on substrate stiffness. **(C)** Experimentally observed cell reversal frequencies in A^+^S^−^ (*pilA^−^*) cells on surfaces with various stiffness tuned by agar concentration (% w/v). Blue circles and the right axis show the number of cells measured for each agar concentration.

Next, we used the model to predict how substrate stiffness affects cell reversal frequency through the connection between the A-motor and the polarity pathway. Previous observations showed that harder substrates induce more intense clustering of A-motors at the cell-substrate interface (Nan et al., 2010). Because clustered A-motors at the substrate interface engage in force generation (Nan et al., 2013; Faure et al., 2016), this phenomenon indicates that harder substrates increase the number of engaged motors. This implication was confirmed by single-molecule tracking of AglR, a protein component of the energy-harvesting unit in A-motors: the number of fast-moving AglR molecules—representing motors that are not engaged in force generation—decreased as substrate stiffness increased (Nan et al., 2013). In light of these experimental observations, in the model we represented substrate stiffness by the motor engagement rate [conversion from an active motor (M_a+_ or M_a−_ in Figure 1) to an engaged motor (M_e+_ or M_e−_ in Figure 1)]. Particularly, a harder substrate corresponds to a higher engagement rate, and vice versa (Figure 3A). We tuned the model parameters such that the fraction of engaged motors falls in a similar range as the experimental measurements on 0.8–5% agar (Nan et al., 2013) (Figure 3A).

Our model predicts that the reversal frequency decreases as the motor engagement rate increases (i.e., as substrate stiffness increases) (Figure 3B). This prediction was validated by the experimental observation in A^+^S^−^ (*pilA^−^*) cells (Figure 3C). Note that as the production of pilin, the subunit of type IV pilus that drives S-motility, was disabled in these mutant cells, motility-mediated mechanosensing can only be attributed to the A-motility machinery, and hence these cells are more closely related to the model than wild type cells.

The above model prediction can be understood in the following way. Recall that MglA binds to the active and engaged motors. An active motor can quickly reach either cell pole [~2.5 s to traverse the typical cell length of 5 μm with the typical speed of 2 μm/s (Nan et al., 2015)]. If it reaches the trailing pole, the motor is inactivated and releases MglA. In contrast, an engaged motor moves towards the trailing pole at a much slower speed [roughly the cell speed, ~2 μm/min, as the engaged motors are nearly stationary relative to the substrate (Mignot et al., 2007; Nan et al., 2010, 2013)]. Therefore, the engaged motors effectively sequester MglA in nonpolar regions. The higher motor engagement rate on a stiffer substrate boosts the number of engaged motors and hence sequesters MglA more strongly away from cell poles. The reduction of MglA at the poles, in turn, decreases the cell reversal frequency (Supplementary Figure S1). Taken together, increased motor engagement on harder substrates sequesters MglA away from cell poles and thus reduces cell reversal frequency.

### Activation of A-motors affects cell reversal frequency

**Figure 4:**
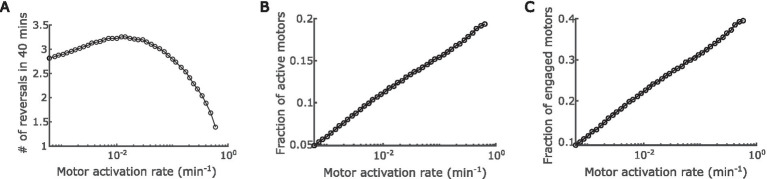
Impact of regulation of A-motor activation. **(A)** Predicted dependence of the cell reversal frequency on the activation rate of A-motors. **(B)** Predicted dependence of the fraction of active A-motors on the motor activation rate. **(C)** Predicted dependence of the fraction of engaged A-motors on the motor activation rate.

Next, we thoroughly explored the effect of A-motor dynamics on cell reversal frequency. The engagement and disengagement of A-motors are reverse reactions, and hence the effects of varying their rates are just inversed. Similar relation is true between activation and deactivation of A-motors. For these reverse reactions, we only need to investigate the effect of one reaction out of a pair. In the last section we explored the effect of engagement rate. Here we focused on the activation rate of A-motors, which refers to the conversion from a diffusive inactive motor (M_i_ in Figure 1) to a directional active motor (M_a+_ or M_a−_ in Figure 1). Interestingly, we found a biphasic relationship between A-motor activation and cell reversal: as the activation rate rises, the cell reversal frequency first increases and then decreases (Figure 4A).

The above prediction can be understood as follows. On the one hand, boosting the A-motor activation rate increases the proportion of active motors (Figure 4B), which enhances directed transport of active (GTP-bound) MglA towards the trailing pole, and thus promotes polarity switching. Note that MglA can also diffuse from one pole to the other, but because most diffusive MglA molecules are inactive (GDP-bound), active MglA cannot be efficiently transported by diffusion. On the other hand, enhanced motor activation also increases the proportion of engaged motors (Figure 4C), because the ratio between active and engaged motors are roughly constant when the engagement rate is fixed (Supplementary Figure S2C). As reasoned in the previous section, the engaged motors effectively sequester MglA in nonpolar regions and consequently reduce the reversal frequency. The effect of increasing active motors dominates when the A-motor activation rate is low, while the effect of increasing engaged motors dominates when A-motor activation rate is high. The transition happens presumably because enhanced transport of active MglA has an immediate influence on the polar MglA/MglB/RomR dynamics, while the sequestration of MglA only has significant impact when a substantial fraction of MglA is sequestered. Together, these amount to a biphasic dependence of cell reversal frequency on the activation rate of A-motors.

### Proper reversal control networks can explain disparities in mechanosensing behaviors in A^+^S^−^ vs. wild type cells

Our model has predicted how mechanosensing regulates cell reversal through A-motility alone, which matches the observation in A^+^S^−^ cells. Different from A^+^S^−^ cells, however, wild type cells that move by both A- and S-motility reverse more frequently on harder surfaces (Zhou and Nan, 2017). This observation suggests that type IV pili that drive S-motility also mediate mechanosensing. However, direct control of cell reversal by the S-motility machinery seems unlikely. Although MglA interacts with certain S-motility proteins, such as FrzS and SgmX (Mercier et al., 2020; Potapova et al., 2020; Bautista et al., 2023), these proteins function downstream of MglA; none of them were reported to transport MglA like the A-motility machinery does or directly regulate its activity. Furthermore, the reversal frequency of cells that move by S-motility alone does not change in response to substrate stiffness (Zhou and Nan, 2017), suggesting that A-motility may be required for S-motility mediated mechanosensing. Therefore, we hypothesize that the A-motility machinery serves as the hub of mechanosensing, and the S-motility machinery only mediates an indirect control of cell reversal through the A-motility machinery.

**Figure 5:**
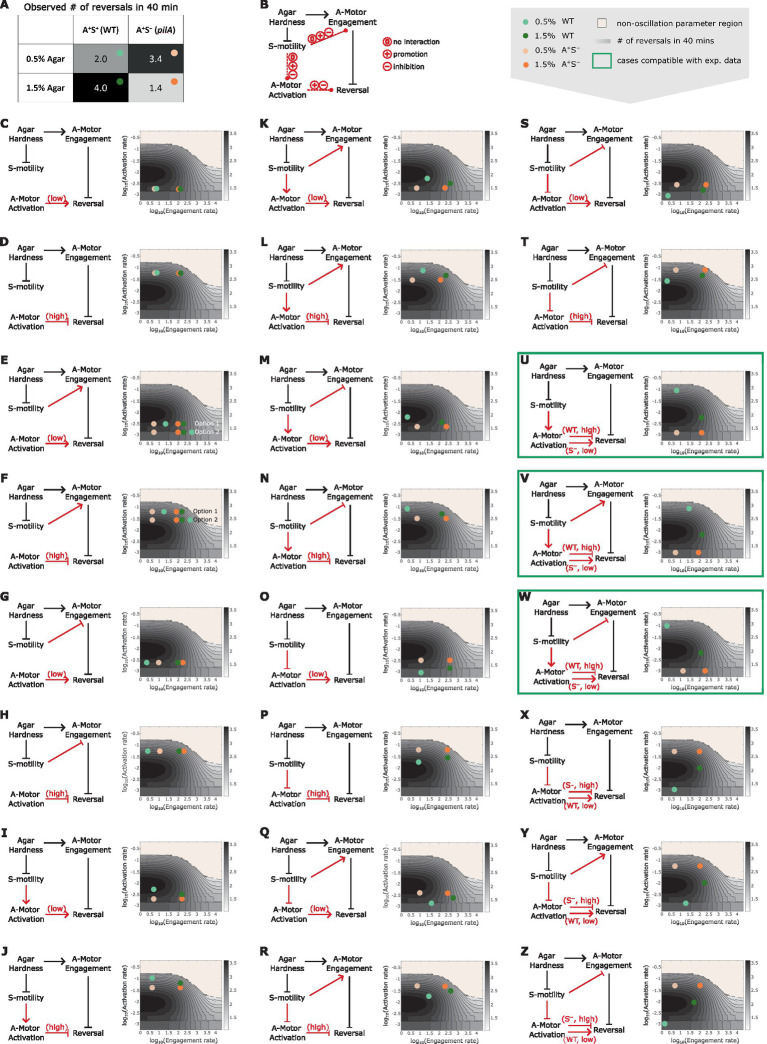
Reversal control networks that reconcile the contrasting mechanosensing responses in wild type vs. A^+^S^−^ cells. **(A)** Experimentally observed cell reversal frequencies of wild type and A^+^S^−^ cells on soft (0.5%) and hard (1.5%) agar surfaces. The observed reversal frequencies are visually represented by the shade of the corresponding cells of the table, where darker colors indicate higher reversal frequencies. **(B)** Summary of all generic reversal control networks in which the S-motility machinery controls cell reversal indirectly through the A-motility machinery. Black arrows: controls of certainty. Red arrows: controls with uncertainty. **(C–Z)** Examination of individual networks for their qualitative compatibility with the experimental data. Representative parameter points for wild type and A^+^S^−^ cells on 0.5 and 1.5% agar surfaces for each network (left) were chosen according to the expected relations among the four cases of interest in terms of their A-motor engagement and activation rates, and are shown on the 2D phase diagram (right, same as Supplementary Figure S2B). Note that due to the higher activity of S-motility on soft substrates, its influence on A-motors is expected to be more pronounced on 0.5% agar compared to 1.5% agar. Hence, the parameter point representing the wild type cell on 0.5% agar (light green dots) must be located in a similar direction relative to the parameter point for the A^+^S^−^ cell on 0.5% agar (light orange dots) as the parameter point for the wild type cell on 1.5% agar (dark green dots) is relative to the parameter point for the A^+^S^−^ cell on 1.5% agar (bright orange dots), except that the former pair of dots (light green vs. light orange) are separated by a greater distance. For example, in panel (K), because S-motility promotes both activation and engagement of A-motors, the dark green and light green dots are located northeast of the bright orange and light orange dots, respectively; but the distance between the light green and light orange dots is larger than that between the dark green and bright orange dots. Green boxes highlight networks that can explain the qualitative relationship among the experimental data points shown in **(A)**.

To test the hypothesis, we combined the predicted dependence of cell reversal frequency on both the engagement and activation rates of A-motors (Supplementary Figure S2), and used the combined model prediction to screen various reversal control networks for those that can qualitatively explain the experimentally observed cell reversal frequencies in wild type and A^+^S^−^ cells on 0.5 and 1.5% agar surfaces (Figure 5A). To focus on our hypothesis, we only examined networks where the S-motility machinery regulates cell reversal indirectly through the A-motility machinery. The candidate networks consist of several certain and uncertain controls. Certain controls include the positive dependence of the A-motor engagement rate on substrate stiffness (Nan et al., 2010), the model-predicted inhibition of cell reversal by A-motor engagement, and the negative dependence of S-motility on substrate stiffness [S-motility is more effective on soft substrates (Shi and Zusman, 1993)] (Figure 5A). Uncertain controls include (i) the possibility that S-motility could influence the engagement and activation of A-motors, each in three possible ways: no effect, promotion, or inhibition (Figure 5A); and (ii) the cell reversal frequency either increases with stronger motor activation in the region of low activation rate, or decreases in the region of high activation rate (Figure 4A and Supplementary Figure S2A). Combination of the above possibilities gave rise to 18 possible networks (Figures 5C–T). Reversal frequencies of the wild type and A^+^S^−^ cells on 0.5 and 1.5% agar surfaces for each network were estimated from the predicted 2D phase diagram for the dependence of cell reversal frequency on the engagement and activation rates of A-motors (same as Supplementary Figure S2A). According to these estimates, none of the 18 networks qualitatively explained the intertwined dependence of cell reversal frequency on substrate stiffness in both genetic backgrounds.

We then considered additional networks in which the wild type and A^+^S^−^ cells reside in different regions of the phase diagram in terms of the A-motor activation rate (Figures 5U–Z). Particularly, if S-motility promotes A-motor activation, then wild type cells should have high rate of A-motor activation and increasing the activation rate in this region inhibits cell reversal (Figures 5U–W). In contrast, due to the lack of S-motility, A^+^S^−^ cells should have low rate of A-motor activation and increasing the activation rate in this region promotes cell reversal (Figures 5U–W). Following the same logic, opposite predictions are yielded if S-motility inhibits A-motor activation, i.e., A-motor activation promotes cell reversal in wild type cells, and inhibits it in A^+^S^−^ cells (Figures 5X–Z). The possibility that S-motility does not affect A-motor activation was not considered, because in this case the wild type and A^+^S^−^ cells cannot have different A-motor activation rates. Among the six additional networks, we found three (Figures 5U–W) that qualitatively reproduced the experimental data on different agar surfaces in both genetic backgrounds (Supplementary Figure S3). In all the three viable networks, S-motility promotes A-motor activation. In contrast, whether and how S-motility regulates A-motor engagement does not affect the qualitative outputs of these networks. In summary, promotion of A-motor activation by S-motility is necessary to reconcile the seemingly contradictory mechanosensing behaviors in wild type vs. A^+^S^−^ cells.

## Discussion

Mechanosensing is an important function of bacteria, which allows them to ‘perceive’ the properties of the surfaces in contact and adjust behaviors accordingly. Through mechanosensing, *M. xanthus* regulates its cell reversal frequency in response to external mechanical cues, such as substrate stiffness and physical contacts with colony mates or prey cells. These responses are crucial for complex pattern formation in *M. xanthus* populations (Igoshin et al., 2001, 2004). Here we developed the first mathematical model for mechanosensing-based reversal control in *M. xanthus* in response to substrate stiffness. The model highlights the interplay between the polarity pathway and the A-motor, particularly incorporating the experimentally established dynamics of A-motors and their binding to polarity regulator MglA (Figure 1). Based on the previously observed intensification of A-motor clustering on hard substrates (Nan et al., 2010), the model uses the A-motor engagement rate to represent substrate stiffness. The model predicts a dependence of cell reversal frequency on substrate stiffness that is consistent with that found in cells that move with A-motility alone (A^+^S^−^) (Figure 3). Furthermore, the model predicts a biphasic dependence of the reversal frequency on the activation rate of A-motors (Figure 4). Finally, we tested the hypothesis that the S-motility machinery mediates reversal control indirectly through the A-motility machinery, and found that an additional promotion of A-motor activation by S-motility can explain why the wild type and A^+^S^−^ cells show opposite responses to substrate stiffness (Zhou and Nan, 2017) (Figure 5). This model prediction awaits future experimental validation. Overall, our model suggests that the A-motility machinery of *M. xanthus* serves as a hub of mechanosensing-based reversal control, which modulates cell reversal in response to environmental mechanical cues.

Note that our model predictions are qualitative, as the model was built upon simplified mechanism of the A-motility machinery and polarity pathway, both of which comprise many molecules that dynamically interact with each other, but the details of these interactions are yet elusive. For instance, the observed spatial dynamics of Agl proteins that constitute the energy-harvesting core of the A-motility machinery and the MreB molecules that constitute the intracellular track for A-motility switch between immotile, directed motion, and diffusion (Nan et al., 2013, 2015; Fu et al., 2018). However, their population distributions do not exhibit three clearly distinct peaks that correspond to these three movement patterns, indicating that the dynamics of the A-motility machinery is more intricate than the three states assumed in the model. Moreover, even though the cast of molecular players constituting the A-motility machinery is increasingly clear (Luciano et al., 2011; Faure et al., 2016; Islam et al., 2023), how exactly A-motors interact with the substrate is not known (Wong et al., 2021; Chen and Nan, 2022). In the model, we resorted to a generic and simplistic assumption that the motor engagement rate depends on substrate stiffness, based on the observed relationship between the motor clustering intensity and substrate stiffness (Nan et al., 2010). These simplifications could cause the quantitative difference between the model prediction and experimental observation, e.g., in Figure 3. Furthermore, for the polarity pathway, we simply adopted the model from (Guzzo et al., 2018). Details of this core regulatory pathway would certainly affect the model prediction quantitatively. As future experiments disclose more mechanistic and quantitative details about these pathways, we will be able to refine our model and make more accurate predictions. On a side note, here we compared the measured reversal frequencies of wild type cells that we previously published (Zhou and Nan, 2017) with those of A^+^S^−^ cells that we collected in this work. This comparison is warranted, because the reversal frequency assay does not involve any labeling or other perturbations to the cell, and hence the results, especially the qualitative trends, are very robust. These data are sufficient for comparison with the qualitative predictions of our current model. With a more precise model in the future, a more tightly controlled experiment and more detailed analysis of the experimental data (e.g., comparing the distribution of reversal frequencies rather than just the average) could become necessary.

In the last part of our work where we theorize about the opposite reversal responses observed in A^+^S^−^ vs. wild type cells, we chose to confine our choices of regulatory networks to those in which S-motility controls mechanosensing indirectly through the A-motility machinery. This assumption was made mainly because *M. xanthus* cells moving by S-motility alone exhibit similar reversal frequency on soft and hard agar surfaces (Zhou and Nan, 2017), suggesting that S-motility probably does not respond to mechanical cues directly. Recently, MglA was found to activate the S-motility machinery at the leading cell pole (Mercier et al., 2020; Potapova et al., 2020; Bautista et al., 2023). A feedback may exist between the S-motility machinery and MglA, which could be incorporated in future revision of the model.

The mechanical cues sensed by *M. xanthus* are more than just substrate stiffness. Previous experiments show that *M. xanthus* cells can also sense physical contacts with colony mates or preys and modulate the frequency and timing of cell reversals accordingly (Hodgkin and Kaiser, 1977; Welch and Kaiser, 2001; Lobedanz and Sogaard-Andersen, 2003; Kaiser and Welch, 2004; Sogaard-Andersen, 2007). Such cellular responses are key to rippling wave formation (Igoshin et al., 2001, 2004), cooperative predation (Berleman et al., 2006, 2008; Keane and Berleman, 2016) and fruiting body formation (Jelsbak and Sogaard-Andersen, 2003; Kaiser and Welch, 2004; Sozinova et al., 2006; Sliusarenko et al., 2007; Thutupalli et al., 2015) in *M. xanthus* populations. Furthermore, exopolysaccharides (EPS), which comprise the majority of the extracellular matrix of *M. xanthus*, inhibit *M. xanthus* reversal in a dosage-dependent manner (Zhou and Nan, 2017). Meanwhile, methylcellulose has the same effect on *M. xanthus* reversal (Zhou and Nan, 2017). As a synthetic polysaccharide that does not exist naturally, methylcellulose is unlikely to trigger chemically specific signals in *M. xanthus*. Hence, the cell probably senses both EPS and methylcellulose mechanically. It is possible that all these mechanosensing behaviors are mediated by the A-motility machinery. For example, the extracellular polysaccharides may promote or mediate engagement of A-motors, which is predicted by our model to inhibit cell reversal (Figure 3).

Mechanoresponses have been studied in various other cellular systems, such as load-dependent recruitment of stator subunits to the bacterial flagellar motor (Nord et al., 2017; Wadhwa et al., 2019), frequency-dependent alignment of muscle cell stress fiber in response to cyclic mechanical stress (Liu et al., 2008; Hsu et al., 2009), and thickening of arterial walls induced by hypertension (Hahn and Schwartz, 2009). While each system employs its own set of molecules for mechanoresponses, certain general principles are shared. Notably, the components responsible for sensing the force are typically force-generating or force-bearing by themselves (Hoffman et al., 2011). Moreover, mechanical forces are often transduced into biochemical signals that influence downstream pathways (Hoffman et al., 2011). These principles are evident in our mechanosensing model. The force-generating A-motility machinery of *M. xanthus* is proposed to transduce the mechanical cues it senses into a cell reversal signal, through its coupling with the reversal regulator MglA. However, unlike the signals in most well studied mechanoresponse mechanisms, the biochemical signal in our model does not arise from force-induced protein conformational changes, but rather from a motor-driven intracellular spatial regulation.

Elucidating the mechanisms of mechanosensing and mechanoresponse is challenging due to the complexity and dynamic nature of these processes. Mathematical modeling provides a useful tool to coherently combine segregated pieces of experimental observations, and generate hypotheses and testable predictions for future experimental studies. Ultimately, integration of modeling and experimentation will provide the best tool to uncover mysteries in bacterial mechanosensing/response and shed light on the intricate interplay between bacterial motility and environmental stimuli.

## Data availability statement

The original contributions presented in the study are included in the article/Supplementary material, and further inquiries can be directed to the corresponding author.

## Author contributions

YC: Data curation, Formal analysis, Investigation, Methodology, Visualization, Writing – original draft, Writing – review & editing, Software. ET: Investigation, Methodology, Writing – original draft. BN: Funding acquisition, Resources, Supervision, Validation, Writing – review & editing, Data curation. JC: Conceptualization, Formal analysis, Funding acquisition, Investigation, Project administration, Resources, Supervision, Validation, Writing – review & editing, Methodology, Visualization.
