# Supplementary material for: Mathematical modeling of mechanosensitive reversal control in Myxococcus xanthus
Source: Front Microbiol. 2024 Jan 8;14:1294631. doi: 10.3389/fmicb.2023.1294631 (PMC10803039; doi:10.3389/fmicb.2023.1294631)
Supplement: Supplementary file 1 [file Data_Sheet_1.pdf]

Supplementary Materials

Mathematical modeling of mechanosensitive reversal control in *Myxococcus xanthus*

Yirui Chen, Elias J. Topo, Beiyan Nan, Jing Chen

Supplementary figures

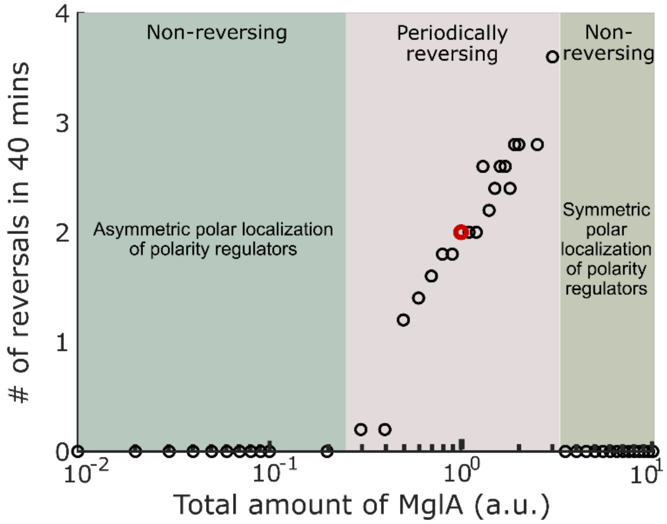

Figure S1. Cell reversal frequency depends on the total amount of MglA in the gated relaxation oscillator model (Guzzo et al., 2018). Red dot: default parameter set.

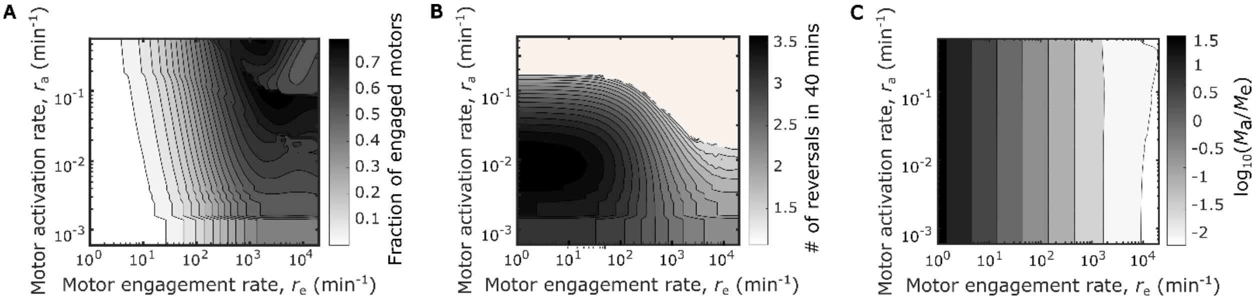

Figure S2. Impact of A-motor engagement rate and activation rate on (A) fraction of engaged A-motors, (B) cell reversal frequency, and (C) ratio between active and engaged A-motors.

	A^+S^+ (WT)	A^+S^- (*pilA*)
**0.5% Agar**	2.5	3.2
**1.5% Agar**	3.3	2.5

Figure S3: Cell reversal frequencies resulting from the selected reversal control network. Simulated data correspond to the case in Figure 5U. The results qualitatively match the experimental data in Figure 5A. Note that the cases given by Figures 5V and 5W involve additional controls and correspondingly additional parameters. With more parameters to adjust, it is trivial to yield similar results in those cases.

Variables and parameters of model

Table S1: Model variables.

Variables	Annotations
M_{i}	Inactive motor
$M_{\mathrm{a}+}$	Active motors moving to the right
$M_{\mathrm{a}-}$	Active motors moving to the left
$M_{\mathrm{e}+}$	Engaged motors moving to the right
$M_{\mathrm{e}-}$	Engaged motors moving to the left
A	MglA
B	MglB
R	RomR
v_{cell}	Cell velocity

Table S2: Superscripts for compartments in the model.

Superscript	Annotations
(l)	Binding sites at left pole
(r)	Binding sites at right pole binding site
(c_n)	$n =$ 1, cytoplasmic domain at left pole<br>$n =$ 6, cytoplasmic domain at right pole<br>$n =$ 2, 3, 4, 5, cytoplasmic domains between poles from left to right

Table S3: Model parameters.

Parameters	Annotations	Values	Source/Reason
D_{Mi}	Diffusion coefficient of inactive motor *	30 min^{-1}	~0.3 $\mu\mathrm{m}^2/\mathrm{s}$ (Fu et al., 2018)
D_{Ma}	Diffusion coefficient of active motor *	30 min^{-1}	~0.3 $\mu\mathrm{m}^2/\mathrm{s}$ (Fu et al., 2018)
D_{Me}	Diffusion coefficient of force-generating motor *	0.1 min^{-1}	<< D_{Ma}
D	Diffusion coefficient of molecules other than the motor *	600 min^{-1}	(Segall et al., 1985;Mullineaux et al., 2006)
r_{a}	Activation rate of motor	0.036 min^{-1}	Varied in this work.
r_{r}	Reversal rate of active motor	120 min^{-1}	~120 min^{-1} (Nan et al., 2015)
r_{e}	Motor engagement rate at focal adhesion sites	6 min^{-1}	Varied in this work.
r_{de}	Motor disengagement rate	10 min^{-1}	

v_M	Velocity of active motor	120 min^{-1}	~60 µm/min (Nan et al., 2015)
v_0	Scaling factor to match *M. xanthus* cell velocity	2 min^{-1}	Typical *M. xanthus* velocity
k_A	Polar binding rate of MglA due to polar RomR *	2400 min^{-1}	(Guzzo et al., 2018)
d_A	Polar unbinding rate of MglA	2 min^{-1}	(Guzzo et al., 2018)
d_AB	Polar unbinding rate of MglA due to polar MglB	400 min^{-1}	(Guzzo et al., 2018)
k_B	Basal polar binding rate of MglB *	12 min^{-1}	(Guzzo et al., 2018)
k_BB	Polar binding rate of MglB due to polar MglB *	180 min^{-1}	(Guzzo et al., 2018)
d_B	Polar unbinding rate of MglB	2.8 min^{-1}	(Guzzo et al., 2018)
d_BA	Polar unbinding rate of MglB due to polar MglA	180 min^{-1}	(Guzzo et al., 2018)
K	Saturation constant for inhibition of polar unbinding of MglB	0.3	(Guzzo et al., 2018)
k_R	Basal polar binding rate of RomR *	0.6 min^{-1}	(Guzzo et al., 2018)
k_RB	Polar binding rate of RomR due to polar MglB *	9 min^{-1}	(Guzzo et al., 2018)
d_R	Polar unbinding rate of RomR	0.2 min^{-1}	(Guzzo et al., 2018)
A_total	Total number of MglA molecules **	10^4	
B_total	Total number of MglB molecules **	10^4	
R_total	Total number of RomR molecules **	10^4	

* Parameter values scaled to construct compartmental model.
** The numbers of protein molecules are not known, and these model parameters are arbitrarily chosen. But they do not affect the results of the model, because the model parameters can be easily rescaled to variations in these numbers.

Model equations

$$v_{\mathrm{cell}} = 2v_0\left(\frac{1}{1+e^{-\sum_n\left(M_{\mathrm{e-}}^{(\mathrm{c}_n)}-M_{\mathrm{e+}}^{(\mathrm{c}_n)}\right)}} - 0.5\right)^{1} \tag{S1}$$

$$H(v_{\mathrm{cell}}) = \begin{cases} 1, & v_{\mathrm{cell}} > 0 \\ 0, & v_{\mathrm{cell}} \le 0 \end{cases} \tag{S2}$$

$$\frac{dM_{\mathrm{i}}^{(\mathrm{c}_1)}}{dt} = D_{\mathrm{Mi}} - r_{\mathrm{a}}\mathrm{M}_{\mathrm{i}}^{(\mathrm{c}_1)}A^{(\mathrm{l})} + \left(M_{\mathrm{a+}}^{(\mathrm{c}_1)} + M_{\mathrm{a-}}^{(\mathrm{c}_1)}\right)\left(d_{\mathrm{A}} + d_{\mathrm{AB}}(\frac{B^{(\mathrm{l})}}{B_{\mathrm{total}}})^2\right) \tag{S3}$$

$$\frac{dM_{\mathrm{i}}^{(\mathrm{c}_n)}}{dt} = D_{\mathrm{Mi}}\left(M_{\mathrm{i}}^{(\mathrm{c}_{n-1})} - 2M_{\mathrm{i}}^{(\mathrm{c}_n)} + M_{\mathrm{i}}^{(\mathrm{c}_{n+1})}\right), \qquad n = 2,3,4,5 \tag{S4}$$

$$\begin{aligned}\frac{dM_{\mathrm{i}}^{(\mathrm{c}_6)}}{dt} = {} & D_{\mathrm{Mi}}\left(M_{\mathrm{i}}^{(\mathrm{c}_5)} - M_{\mathrm{i}}^{(\mathrm{c}_6)}\right) - r_{\mathrm{a}}M_{\mathrm{i}}^{(\mathrm{r})}A^{(\mathrm{r})} \\ & + \left(M_{\mathrm{a+}}^{(\mathrm{c}_6)} + M_{\mathrm{a-}}^{(\mathrm{c}_6)}\right)\left(d_A + d_{AB}(\frac{B^{(\mathrm{r})}}{B_{\mathrm{total}}})^2\right)\end{aligned} \tag{S5}$$

$$\begin{aligned}\frac{dM_{\mathrm{a+}}^{(\mathrm{c}_1)}}{dt} = {} & D_{\mathrm{Ma}}\left(M_{\mathrm{a+}}^{(\mathrm{c}_2)} - M_{\mathrm{a+}}^{(\mathrm{c}_1)}\right) - v_{\mathrm{M}}\left(M_{\mathrm{a+}}^{(\mathrm{c}_1)} - M_{\mathrm{a-}}^{(\mathrm{c}_1)}\right) + r_{\mathrm{r}}\left(M_{\mathrm{a-}}^{(\mathrm{c}_1)} - M_{\mathrm{a+}}^{(\mathrm{c}_1)}\right) \\ & - \mathrm{r_e}M_{\mathrm{a+}}^{(\mathrm{c}_1)} + \mathrm{r_{de}}M_{\mathrm{e+}}^{(\mathrm{c}_1)} + H(v_{\mathrm{cell}})|v_{\mathrm{cell}}|M_{\mathrm{e+}}^{(\mathrm{c}_1)} + r_{\mathrm{a}}M_{\mathrm{i}}^{(\mathrm{c}_1)}A^{(\mathrm{l})} \\ & - \left(d_{\mathrm{A}} + d_{\mathrm{AB}}(\frac{B^{(\mathrm{l})}}{B_{\mathrm{total}}})^2\right)M_{\mathrm{a+}}^{(\mathrm{c}_1)}\end{aligned} \tag{S6}$$

$$\begin{aligned}\frac{dM_{\mathrm{a+}}^{(\mathrm{c}_n)}}{dt} = {} & D_{\mathrm{Ma}}\left(M_{\mathrm{a+}}^{(\mathrm{c}_{n-1})} - 2M_{\mathrm{a+}}^{(\mathrm{c}_n)} + M_{\mathrm{a+}}^{(\mathrm{c}_{n+1})}\right) + v_{\mathrm{M}}\left(M_{\mathrm{a+}}^{(\mathrm{c}_{n-1})} - M_{\mathrm{a+}}^{(\mathrm{c}_n)}\right) \\ & + r_{\mathrm{r}}\left(M_{\mathrm{a-}}^{(\mathrm{c}_n)} - M_{\mathrm{a+}}^{(\mathrm{c}_n)}\right) - \mathrm{r_e}M_{\mathrm{a+}}^{(\mathrm{c}_n)} + \mathrm{r_{de}}M_{\mathrm{e+}}^{(\mathrm{c}_n)}, \qquad n = 2,3,4,5\end{aligned} \tag{S7}$$

$$\begin{aligned}\frac{dM_{\mathrm{a+}}^{(\mathrm{c}_6)}}{dt} = {} & D_{\mathrm{Ma}}\left(M_{\mathrm{a+}}^{(\mathrm{c}_5)} - M_{\mathrm{a+}}^{(\mathrm{c}_6)}\right) + v_{\mathrm{M}}\left(M_{\mathrm{a+}}^{(\mathrm{c}_5)} - M_{\mathrm{a+}}^{(\mathrm{c}_6)}\right) + r_{\mathrm{r}}\left(M_{\mathrm{a-}}^{(\mathrm{c}_6)} - M_{\mathrm{a+}}^{(\mathrm{c}_6)}\right) \\ & - \mathrm{r_e}M_{\mathrm{a+}}^{(\mathrm{c}_6)} + \mathrm{r_{de}}M_{\mathrm{e+}}^{(\mathrm{c}_6)} + \left(1 - H(v_{\mathrm{cell}})\right)|v_{\mathrm{cell}}|M_{\mathrm{e+}}^{(\mathrm{c}_6)} \\ & - \left(d_{\mathrm{A}} + d_{\mathrm{AB}}(\frac{B^{(\mathrm{r})}}{B_{\mathrm{total}}})^2\right)M_{\mathrm{a+}}^{(\mathrm{c}_6)}\end{aligned} \tag{S8}$$

$$\begin{aligned}\frac{dM_{\mathrm{a-}}^{(\mathrm{c}_1)}}{dt} = {} & D_{\mathrm{Ma}}\left(M_{\mathrm{a-}}^{(\mathrm{c}_2)} - M_{\mathrm{a-}}^{(\mathrm{c}_1)}\right) + v_{\mathrm{M}}\left(M_{\mathrm{a-}}^{(\mathrm{c}_2)} - M_{\mathrm{a-}}^{(\mathrm{c}_1)}\right) + r_{\mathrm{r}}\left(M_{\mathrm{a+}}^{(\mathrm{c}_1)} - M_{\mathrm{a+}}^{(\mathrm{c}_1)}\right) \\ & - \mathrm{r_e}M_{\mathrm{a-}}^{(\mathrm{c}_1)} + \mathrm{r_{de}}M_{\mathrm{e-}}^{(\mathrm{c}_1)} + H(v_{\mathrm{cell}})|v_{\mathrm{cell}}|M_{\mathrm{e-}}^{(\mathrm{c}_1)} \\ & - \left(d_A + d_{AB}(\frac{B^{(l)}}{B_{\mathrm{total}}})^2\right)M_{\mathrm{a-}}^{(\mathrm{c}_1)}\end{aligned} \tag{S9}$$

---

[1] The cell velocity was measured to be nearly constant in the range of agar densities relevant to this work (Tchoufag et al., 2019). These experimental data indicate a nonlinear relation between cell velocity and the number of engaged focal adhesion complexes. To account for this nonlinear relationship and constant velocity over various agar densities, we assumed a simple sigmoidal dependence of the cell velocity on the difference of number of motors traveling in the positive versus the negative directions.

$$\frac{dM_{\mathrm{a-}}^{(\mathrm{c}_n)}}{dt} = D_{\mathrm{Ma}}\left(M_{\mathrm{a-}}^{(\mathrm{c}_{n-1})} - 2M_{\mathrm{a-}}^{(\mathrm{c}_n)} + M_{\mathrm{a-}}^{(\mathrm{c}_{n+1})}\right) + v_{\mathrm{M}}\left(M_{\mathrm{a-}}^{(\mathrm{c}_{n+1})} - M_{\mathrm{a-}}^{(\mathrm{c}_n)}\right) + r_{\mathrm{r}}\left(M_{\mathrm{a+}}^{(\mathrm{c}_n)} - M_{\mathrm{a-}}^{(\mathrm{c}_n)}\right) - r_{\mathrm{e}}M_{\mathrm{a-}}^{(\mathrm{c}_n)} + r_{\mathrm{de}}M_{\mathrm{e-}}^{(\mathrm{c}_n)}, \qquad n = 2,3,4,5 \tag{S10}$$

$$\frac{dM_{\mathrm{a-}}^{(\mathrm{c}_6)}}{dt} = D_{\mathrm{Ma}}\left(M_{\mathrm{a-}}^{(\mathrm{c}_5)} - M_{\mathrm{a-}}^{(\mathrm{c}_6)}\right) - v_{\mathrm{M}}\left(M_{\mathrm{a-}}^{(\mathrm{c}_6)} - M_{\mathrm{a+}}^{(\mathrm{c}_6)}\right) + r_{\mathrm{r}}\left(M_{\mathrm{a+}}^{(\mathrm{c}_6)} - M_{\mathrm{a-}}^{(\mathrm{c}_6)}\right) - r_{\mathrm{e}}M_{\mathrm{a-}}^{(\mathrm{c}_6)} + r_{\mathrm{de}}M_{\mathrm{e-}}^{(\mathrm{c}_6)} + \left(1 - H(v_{\mathrm{cell}})\right)|v_{\mathrm{cell}}|M_{\mathrm{e-}}^{(\mathrm{c}_6)} + r_{\mathrm{a}}M_{\mathrm{i}}^{(\mathrm{c}_6)}A^{(\mathrm{r})} - \left(d_A + d_{AB}\left(\frac{B^{(\mathrm{r})}}{B_{\mathrm{total}}}\right)^2\right)M_{\mathrm{a-}}^{(\mathrm{c}_6)} \tag{S11}$$

$$\frac{dM_{\mathrm{e+}}^{(\mathrm{c}_1)}}{dt} = D_{\mathrm{Me}}\left(M_{\mathrm{e+}}^{(\mathrm{c}_2)} - M_{\mathrm{e+}}^{(\mathrm{c}_1)}\right) - \left(1 - H(v_{\mathrm{cell}})\right)|v_{\mathrm{cell}}|M_{\mathrm{e+}}^{(\mathrm{c}_1)} + H(v_{\mathrm{cell}})|v_{\mathrm{cell}}|\left(M_{\mathrm{e+}}^{(\mathrm{c}_2)} - M_{\mathrm{e+}}^{(\mathrm{c}_1)}\right) + r_{\mathrm{e}}M_{\mathrm{a+}}^{(\mathrm{c}_1)} - r_{\mathrm{de}}M_{\mathrm{a+}}^{(\mathrm{c}_1)} \tag{S12}$$

$$\frac{dM_{\mathrm{e+}}^{(\mathrm{c}_n)}}{dt} = D_{\mathrm{Me}}\left(M_{\mathrm{e+}}^{(\mathrm{c}_{n-1})} - 2M_{\mathrm{e+}}^{(\mathrm{c}_n)} + M_{\mathrm{e+}}^{(\mathrm{c}_{n+1})}\right) + \left(1 - H(v_{\mathrm{cell}})\right)|v_{\mathrm{cell}}|\left(M_{\mathrm{e+}}^{(\mathrm{c}_{n-1})} - M_{\mathrm{e+}}^{(\mathrm{c}_n)}\right) + H(v_{\mathrm{cell}})|v_{\mathrm{cell}}|\left(M_{\mathrm{e+}}^{(\mathrm{c}_{n+1})} - M_{\mathrm{e+}}^{(\mathrm{c}_n)}\right) + r_{\mathrm{e}}M_{\mathrm{a+}}^{(\mathrm{c}_n)} - r_{\mathrm{de}}M_{\mathrm{e+}}^{(\mathrm{c}_n)}, \quad n = 2,3 \tag{S13}$$

$$\frac{dM_{\mathrm{e+}}^{(\mathrm{c}_6)}}{dt} = D_{\mathrm{Me}}\left(M_{\mathrm{e+}}^{(\mathrm{c}_5)} - M_{\mathrm{e+}}^{(\mathrm{c}_6)}\right) + \left(1 - H(v_{\mathrm{cell}})\right)|v_{\mathrm{cell}}|\left(M_{\mathrm{e+}}^{(\mathrm{c}_5)} - M_{\mathrm{e+}}^{(\mathrm{c}_6)}\right) - H(v_{\mathrm{cell}})|v_{\mathrm{cell}}|M_{\mathrm{e+}}^{(\mathrm{c}_6)} + r_{\mathrm{e}}M_{\mathrm{a+}}^{(\mathrm{c}_6)} - r_{\mathrm{de}}M_{\mathrm{e+}}^{(\mathrm{c}_6)} \tag{S14}$$

$$\frac{dM_{\mathrm{e-}}^{(\mathrm{c}_1)}}{dt} = D_{\mathrm{Me}}\left(M_{\mathrm{e-}}^{(\mathrm{c}_2)} - M_{\mathrm{e-}}^{(\mathrm{c}_1)}\right) - \left(1 - H(v_{\mathrm{cell}})\right)|v_{\mathrm{cell}}|M_{\mathrm{e-}}^{(\mathrm{c}_1)} + H(v_{\mathrm{cell}})|v_{\mathrm{cell}}|(M_{\mathrm{e-}}^{(\mathrm{c}_2)} - M_{\mathrm{e-}}^{(\mathrm{c}_1)}) + r_{\mathrm{e}}M_{\mathrm{a-}}^{(\mathrm{c}_1)} - r_{\mathrm{de}}M_{\mathrm{e-}}^{(\mathrm{c}_1)} \tag{S15}$$

$$\frac{dM_{\mathrm{e-}}^{(\mathrm{c}_n)}}{dt} = D_{\mathrm{Me}}\left(M_{\mathrm{e-}}^{(\mathrm{c}_{n-1})} - 2M_{\mathrm{e-}}^{(\mathrm{c}_n)} + M_{\mathrm{e-}}^{(\mathrm{c}_{n+1})}\right) + \left(1 - H(v_{\mathrm{cell}})\right)|v_{\mathrm{cell}}|(M_{\mathrm{e-}}^{(\mathrm{c}_{n-1})} - M_{\mathrm{e-}}^{(\mathrm{c}_n)}) + H(v_{\mathrm{cell}})|v_{\mathrm{cell}}|(M_{\mathrm{e-}}^{(\mathrm{c}_{n+1})} - M_{\mathrm{e-}}^{(\mathrm{c}_n)}) + r_{\mathrm{e}}M_{\mathrm{a-}}^{(\mathrm{c}_n)} - r_{\mathrm{de}}M_{\mathrm{e-}}^{(\mathrm{c}_n)}, \qquad n = 2,3,4,5 \tag{S16}$$

$$\frac{dM_{\mathrm{e-}}^{(\mathrm{c}_6)}}{dt} = D_{\mathrm{Me}}\left(M_{\mathrm{e-}}^{(\mathrm{c}_5)} - M_{\mathrm{e-}}^{(\mathrm{c}_6)}\right) + \left(1 - H(v_{\mathrm{cell}})\right)|v_{\mathrm{cell}}|\left(M_{\mathrm{e-}}^{(\mathrm{c}_5)} - M_{\mathrm{e-}}^{(\mathrm{c}_6)}\right) - H(v_{\mathrm{cell}})|v_{\mathrm{cell}}|M_{\mathrm{e-}}^{(\mathrm{c}_6)} + r_{\mathrm{e}}M_{\mathrm{a-}}^{(\mathrm{c}_6)} - r_{\mathrm{de}}M_{\mathrm{e-}}^{(\mathrm{c}_6)} \tag{S17}$$

$$\frac{dA^{(\mathrm{l})}}{dt} = \frac{k_{\mathrm{A}}A^{(\mathrm{c}_1)}R^{(\mathrm{l})}}{R_{\mathrm{total}}} - d_{\mathrm{A}}A^{(\mathrm{l})} - d_{\mathrm{AB}}A^{(\mathrm{l})}\left(\frac{B^{(\mathrm{l})}}{B_{\mathrm{total}}}\right)^2 - r_{\mathrm{a}}M_{\mathrm{i}}^{(\mathrm{c}_1)}A^{(\mathrm{l})} + \left(M_{\mathrm{a+}}^{(\mathrm{c}_1)} + M_{\mathrm{a-}}^{(\mathrm{c}_1)}\right)\left(d_A + d_{AB}\left(\frac{B^{(l)}}{B_{\mathrm{total}}}\right)^2\right) \tag{S18}$$

$$\frac{dA^{(\mathrm{c}_1)}}{dt} = \frac{-k_{\mathrm{A}}A^{(\mathrm{c}_1)}R^{(\mathrm{l})}}{R_{\mathrm{total}}} + d_{\mathrm{A}}A^{(\mathrm{l})} + d_{\mathrm{AB}}A^{(\mathrm{l})}\left(\frac{B^{(\mathrm{l})}}{B_{\mathrm{total}}}\right)^2 + D(A^{(\mathrm{c}_2)} - A^{(\mathrm{c}_1)}) \tag{S19}$$

$$\frac{dA^{(\mathrm{c}_n)}}{dt} = D\left(A^{(\mathrm{c}_{n-1})} - 2A^{(\mathrm{c}_n)} + A^{(\mathrm{c}_{n+1})}\right), \qquad n = 2,3,4,5 \tag{S20}$$

$$\frac{dA^{(c_6)}}{dt} = \frac{-k_A A^{(c_6)} R^{(r)}}{R_{total}} + d_A A^{(r)} + d_{AB} A^{(r)} (\frac{B^{(r)}}{B_{total}})^2 + D(A^{(c_5)} - A^{(c_6)}) \tag{S21}$$

$$\frac{dA^{(r)}}{dt} = \frac{k_A A^{(c_6)} R^{(r)}}{R_{total}} - d_A A^{(r)} - d_{AB} A^{(r)} (\frac{B^{(r)}}{B_{total}})^2 - r_a M_i^{(c_6)} A^{(r)} + \left(M_{a+}^{(c_6)} + M_{a-}^{(c_6)}\right)\left(d_A + d_{AB} (\frac{B^{(r)}}{B_{total}})^2\right) \tag{S22}$$

$$\frac{dB^{(l)}}{dt} = B^{(c_1)}(k_B + \frac{k_{BB} B^{(l)}}{B_{total}}) - d_B \left(\frac{K}{\frac{B^{(l)}}{B_{total}} + K}\right) B^{(l)} - \frac{d_{BA} A^{(l)} \left(B^{(l)}\right)^2}{A_{total} B_{total}} \tag{S23}$$

$$\frac{dB^{(c_1)}}{dt} = -B^{(c_1)}(k_B + \frac{k_{BB} B^{(l)}}{B_{total}}) + d_B \left(\frac{K}{\frac{B^{(l)}}{B_{total}} + K}\right) B^{(l)} + \frac{d_{BA} A^{(l)} \left(B^{(l)}\right)^2}{A_{total} B_{total}} + D(B^{(c_2)} - B^{(c_1)}) \tag{S24}$$

$$\frac{dB^{(c_n)}}{dt} = D(B^{(c_{n-1})} - 2B^{(c_n)} + B^{(c_{n+1})}), \qquad n = 2,3,4,5 \tag{S25}$$

$$\frac{dB^{(c_6)}}{dt} = -B^{(c_6)}(k_B + \frac{k_{BB} B^{(r)}}{B_{total}}) + d_B \left(\frac{K}{\frac{B^{(r)}}{B_{total}} + K}\right) B^{(r)} + \frac{d_{BA} A^{(r)} \left(B^{(r)}\right)^2}{A_{total} B_{total}} + D(B^{(c_5)} - B^{(c_6)}) \tag{S26}$$

$$\frac{dB^{(r)}}{dt} = B^{(c_6)}(k_B + \frac{k_{BB} B^{(r)}}{B_{total}}) - d_B \left(\frac{K}{\frac{B^{(r)}}{B_{total}} + K}\right) B^{(r)} - \frac{d_{BA} A^{(r)} \left(B^{(r)}\right)^2}{A_{total} B_{total}} \tag{S27}$$

$$\frac{dR^{(l)}}{dt} = R^{(c_1)}(k_R + \frac{k_{RB} B^{(l)}}{B_{total}}) - d_R R^{(l)} \tag{S28}$$

$$\frac{dR^{(c_1)}}{dt} = -R^{(c_1)}(k_R + \frac{k_{RB} B^{(l)}}{B_{total}}) + d_R R^{(l)} + D(R^{(c_2)} - R^{(c_1)}) \tag{S29}$$

$$\frac{dR^{(c_n)}}{dt} = D\left(R^{(c_{n-1})} - 2R^{(c_n)} + R^{(c_{n+1})}\right), \qquad n = 2,3,4,5 \tag{S30}$$

$$\frac{dR^{(c_6)}}{dt} = -R^{(c_6)}(k_R + \frac{k_{RB} B^{(r)}}{B_{total}}) + d_R R^{(r)} + D(R^{(c_5)} - R^{(c_6)}) \tag{S31}$$

$$\frac{dR^{(r)}}{dt} = R^{(c_6)}(k_R + \frac{k_{RB} B^{(r)}}{B_{total}}) - d_R R^{(r)} \tag{S32}$$

Eqs. (S1)~(S32) were simulated using the ode15s solver in MATLAB.

Experimental methods

Reversal frequency was calculated on CYE (10 mM MOPS pH 7.6, 1% (w/v) Bacto™ casitone (BD Biosciences), 0.5% yeast extract, and 8 mM $MgSO_4$) plates containing various concentrations of agar (in w/v). For each strain and condition, 20-min time-lapse videos were recorded with a ZEISS AXIOTM microscope and a ZEISS AxioCamTM MRm camera at 20-s intervals. For each strain and condition, cells were diluted to the point where cells did not contact one another.

Supplementary references

Fu, G., Bandaria, J.N., Le Gall, A.V., Fan, X., Yildiz, A., Mignot, T., Zusman, D.R., and Nan, B. (2018). MotAB-like machinery drives the movement of MreB filaments during bacterial gliding motility. *Proceedings of the National Academy of Sciences* 115**,** 2484-2489.

Guzzo, M., Murray, S.M., Martineau, E., Lhospice, S., Baronian, G., My, L., Zhang, Y., Espinosa, L., Vincentelli, R., Bratton, B.P., Shaevitz, J.W., Molle, V., Howard, M., and Mignot, T. (2018). A gated relaxation oscillator mediated by FrzX controls morphogenetic movements in Myxococcus xanthus. *Nat Microbiol* 3**,** 948-959.

Mullineaux, C.W., Nenninger, A., Ray, N., and Robinson, C. (2006). Diffusion of green fluorescent protein in three cell environments in Escherichia coli. *J Bacteriol* 188**,** 3442-3448.

Nan, B., Bandaria, J.N., Guo, K.Y., Fan, X., Moghtaderi, A., Yildiz, A., and Zusman, D.R. (2015). The polarity of myxobacterial gliding is regulated by direct interactions between the gliding motors and the Ras homolog MglA. *Proc Natl Acad Sci U S A* 112**,** E186-193.

Segall, J.E., Ishihara, A., and Berg, H.C. (1985). Chemotactic signaling in filamentous cells of Escherichia coli. *J Bacteriol* 161**,** 51-59.

Tchoufag, J., Ghosh, P., Pogue, C.B., Nan, B., and Mandadapu, K.K. (2019). Mechanisms for bacterial gliding motility on soft substrates. *Proc Natl Acad Sci U S A* 116**,** 25087-25096.
